# Effects of contralesional robot-assisted hand training in patients with unilateral spatial neglect following stroke: a case series study

**DOI:** 10.1186/1743-0003-11-160

**Published:** 2014-12-05

**Authors:** Valentina Varalta, Alessandro Picelli, Cristina Fonte, Giulia Montemezzi, Elisabetta La Marchina, Nicola Smania

**Affiliations:** Neuromotor and Cognitive Rehabilitation Research Center, Department of Neurological and Movement Sciences, University of Verona, Verona, CA Italy, P.le L.A. Scuro, 10, 37134 Italy; Neurological Rehabilitation Unit, Azienda Ospedaliera Universitaria Integrata, Verona, Italy

**Keywords:** Rehabilitation, Perceptual disorders, Upper extremity

## Abstract

**Background:**

A reduction of hemispatial neglect due to stroke has been associated with activation of the contralesional hand in the contralesional hemispace. Robot-assisted upper limb training was found to effectively improve paretic arm function in stroke patients. To date no proof of concept of robot-assisted hemispatial neglect therapy has been reported in literature. This study aimed to determine whether robot-assisted left (contralesional) hand activation alone could lead to an improvement in hemispatial neglect following stroke.

**Methods:**

Three stroke patients with right brain injury underwent a 2-week training program of robotic left hand activation with the Gloreha® hand rehabilitation glove, which provides repetitive, passive mobilization of the fingers. Outcomes were assessed using the Line Crossing test, the Bells test, the Sentence Reading test, the Saccadic Training, the Sustained Attention to Response Task, and the Purdue Pegboard test.

**Results:**

Changes were observed after treatment as follows. Line Crossing test: all patients showed improved performance (6.7%, 89.5% and 80% increase in lines crossed) with two patients reaching normal performance levels. Bells test: one patient improved performance (50% increase), while one patient showed no change and one patient declined (−10.3% change); no patient reached normal performance levels. Sentence Reading test: all patients showed improved performance (800%, 57.1% and 42.9% increase in number of sentences read) with no patient reaching normal performance level. Saccadic Training: all patients showed improved performance (−62.8%, −15.5% and −9.7% change of the left hemifield reaction time). Sustained Attention to Response Task: all patients showed improved performance (−20.5%, −5.8% and −10% change of the reaction time) with two patients reducing incorrect responses (−42.9% and −73.3%) and one patient increasing them (9.1%). Purdue Pegboard test: all patients showed improved performance (100%, 27.3% and 75% change in the left + right + both hands sub-item score).

**Conclusions:**

Some caution is warranted when interpreting our results, as the responses to the intervention were variable and might have been due to a placebo effect or fluctuating clinical conditions. However, robot-assisted hemispatial neglect therapy might be useful in stroke patients. Larger-scale investigations are needed to confirm our preliminary findings.

**Electronic supplementary material:**

The online version of this article (doi:10.1186/1743-0003-11-160) contains supplementary material, which is available to authorized users.

## Introduction

Hemispatial neglect is a common post-stroke syndrome involving the right brain hemisphere (inferior frontal gyrus, precentral gyrus, postcentral gyrus, superior temporal gyrus, middle temporal gyrus, middle occipital gyrus, insula, and surrounding white matter) [1–3]. Patients suffering from hemispatial neglect consequent to stroke have been described to fail to report or respond or be aware of stimuli located contralateral to the brain lesion [1–6].

Therapeutic interventions to reduce the level of impairment and disability in such patients [3, 7–9] have been found to improve the execution of visuospatial tasks during active movement of the left (contralesional) hand in the homologous hemispace [10–13] or after specific training with limb activation treatment in combination with other rehabilitative approaches to hemispatial neglect [14]. Furthermore, a reduction in hemispatial neglect has been observed during passive left (contralesional) hand movements in the homologous hemispace [15]. Given the high incidence of upper limb paresis associated with (and exacerbated by) the neglect syndrome, the positive effect of passive limb movement on hemispatial neglect is particularly interesting from a rehabilitative point of view [16]. Indeed, following their observations of the effects of functional electrical stimulation (FES) on the neglect syndrome, Eskes & Butler suggested that intentional motor programming is not necessary to improve attention to the left hemispace in such patients [16].

The effects of contralesional limb activation may be interpreted according to the premotor theory of spatial attention [17] that posits the notion that, because the brain’s attention and motor circuits are closely linked, somatosensory activation in the contralesional space through the use of limb activation treatment stimulates the neural networks subserving space representation and thus improves the conscious perception of stimuli in the contralateral hemispace. Hence, movements of the contralesional limb may induce activation of the damaged hemisphere sufficiently to reduce the inhibitory competition from the undamaged hemisphere [18].

There is growing interest in the use of robotic devices in neurological rehabilitation. Robot-assisted upper limb training has been found to safely and effectively improve paretic arm function and activities of daily living in patients with stroke [19, 20]. To the best of our knowledge, no proof of concept of robot-assisted hemispatial neglect therapy has been reported in the neuropsychological rehabilitation literature. Therefore, and building on previous work showing the efficacy of limb activation treatment, we carried out this case series study to determine whether robot-assisted left (contralesional) hand activation alone could lead to an improvement in hemispatial neglect in three patients with stroke.

## Methods

Three adult, right-handed, subjects with right-hemisphere brain damage due to first-ever ischemic stroke were recruited from those referred to the outpatient services of the Neurological Rehabilitation Unit of the Azienda Ospedaliera Universitaria Integrata, Verona, Italy. The enrolment period was from March to August 2012. Subjects were screened at the baseline visit, which included physical and neurological examination and assessment according to the European Stroke Scale [21].

Inclusion criteria were: diagnosis of stroke as documented by a computerized tomography scan or magnetic resonance imaging; age >18 years; right brain damage; Mini Mental State Examination score >24; presence of hemispatial neglect diagnosed by pathological performance on at least two of the following tests: Line Crossing test [22], Bells test [23], and Sentence Reading test [24].

Exclusion criteria were: left upper limb sensory deficit; visual impairment due to cataracts, diabetic retinopathy, glaucoma or hemianopia documented by clinical history; psychotic disorders; alcoholism; drug abuse; aphasia; modified Ashworth scale score for left hand muscle tone >2 [25]; other orthopedic conditions involving the left upper limb; other neurological conditions involving cognitive functions.

All subjects gave their written informed consent to participate in the study. The study was carried out according to the Declaration of Helsinki and approved by the local Ethics Committee. Subjects were asked not to participate in any type of rehabilitation in the 3 months up to the start of the study nor undergo any form of training other than that scheduled in the study protocol.

### Intervention

**Figure 1:**
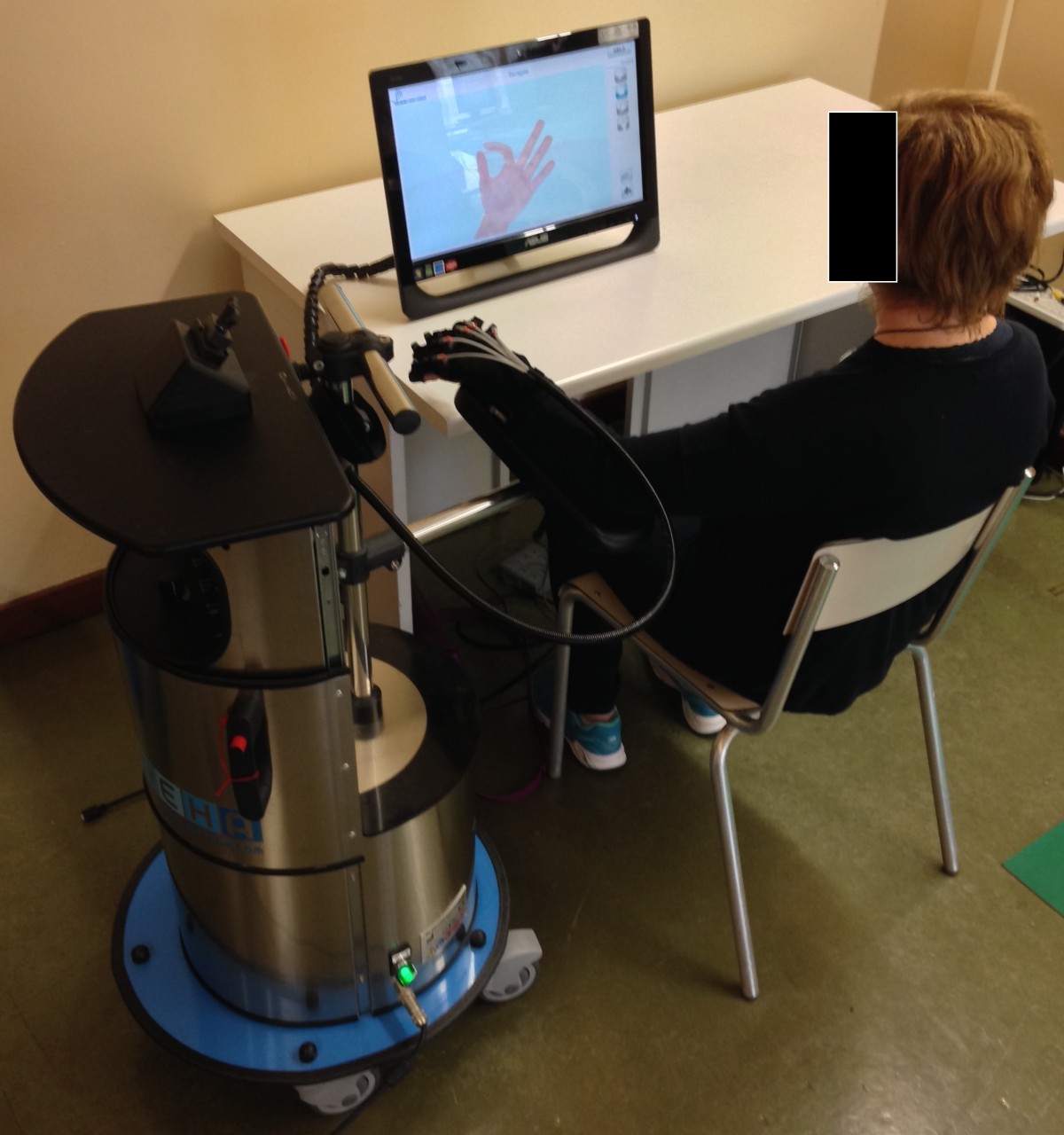
**Robot-assisted hand training.**

The training program consisted of 10 sessions, each lasting 30 minutes including rest periods, 5 days a week (from Monday to Friday) for 2 consecutive weeks. Robot-assisted hand training was performed with the Gloreha® (Idrogenet srl, Lumezzane, Italy) hand rehabilitation glove that provides computer-controlled, repetitive, passive mobilization of the fingers. As shown in Figure 1, the subjects are seated at a height-adjustable support with their elbow bent at 90° and their left hand inserted in a Lycra® glove. Artificial tendons incorporated in the glove move the fingers and the subjects’ hand movements are visualized as a real-time 3D animation on a computer monitor connected to the device.

Each training session consisted of six parts with 1 minute of rest in between. Each session started with a sequence of digital joint flexion/extension exercises (from the thumb to the fifth finger) for 5 minutes (10 repetitions) followed by 5 minutes (15 repetitions) of a number sequence (from one to five). A sequence of opposition movements (from the second to the fifth finger) was then performed for 2 minutes (6 repetitions), followed by 5 minutes (25 repetitions) of a sequence of wave-like finger movements. Finally, a sequence of fist opening/closing was performed for 3 minutes (12 repetitions), followed by 5 minutes (12 repetitions) of a random sequence of digital joint motion. During the first week of treatment, the subjects had to watch the monitor in front of them and name the fingers being moved throughout the training session, while during the second week of treatment they had to name the moving fingers with their eyes closed. The same therapist (G.M.) conducted all training sessions.

### Testing procedures

Subjects were evaluated at baseline (1 week before the first training session) and at endpoint (1 week after the last training session). The same examiner (V.V.) evaluated all subjects.

#### Outcome measures

The Line Crossing test is a visual search task that examines the ability to locate and cancel target stimuli in the neglected hemispace [22]. Here, 40 lines each 25 mm in length were drawn in an apparently random manner on a 200 mm by 260 mm sheet of paper. The total number of lines crossed is recorded. The maximum score is 36 (18 left, 18 right). The 4 lines in the central column are not scored. An omission of 2 lines is considered a pathologic performance [22].

The Bells test allows for qualitative and quantitative assessment of hemispatial neglect in the near extra-personal space. It requires circling 35 targets (bells) embedded within 280 distractors (horses, guitars, houses, etc.) on a 280 mm by 215 mm sheet of paper placed in the centre of patient’s field of view [23]. The spatial distribution of the target figures is arranged so that 5 bells are located in 7 equally sized columns. The total number of circled bells is recorded. A difference of 5 bells omitted between the right and the left side indicates hemispatial neglect [23].

The Sentence Reading test examines the ability to read sentences written from left to right [24]. It consists of 12 phrases, one per sheet, of different lengths (patients with hemispatial neglect usually read sentences from the centre of the sheet whilst neglecting the left half of the phrase). One point is assigned for each correctly read sentence. The maximum score is 12 (a lower score indicates the presence of hemispatial neglect) [24].

The Saccadic Training (subtest of the RehaCom® software) was used to evaluate the reaction time for right and left stimuli [26]. This was because reaction time has been found to yield important information about visuospatial attention in patients with hemispatial neglect [27]. During the evaluation, the subjects were seated in front of a 17” monitor, which showed a sky over a city with a white moon (the focus point) in the centre of the sky. The subjects had to identify the flying objects that appeared in the sky and confirm their appearance by pressing the appropriate key (left or right arrow) on a panel. The reaction time for each stimulus was recorded and the difference between right and left stimuli was computed.

The Sustained Attention to Response Task is a reaction time task for sustained attention and cognitive control that evaluates the ability to sustain attention with a response-suppression element [28]. During this test, sequences of digits 1–9 are visually presented (at a rate of 1 every 2 seconds) in random order up to 25 times to the patient, who has to respond as quickly as possible to each digit except for the third digit. The reaction times are averaged and recorded as well as the number of incorrect responses [28].

The Purdue Pegboard test was used to assess gross movement of the arm, hand and fingers, as well as fingertip dexterity [29, 30]. It uses a board with 4 cups across the top and 2 vertical rows of 25 small holes down the centre. The 2 outside cups contain 25 pins each; the cup to the immediate left contains 40 washers and the cup to the immediate right of the centre contains 20 collars. The Purdue Pegboard test consists of 5 sub-tests as follows: right hand (patients use their right hand to place as many pins as possible down the row within 30 seconds); left hand (patients use their left hand to place as many pins as possible down the row within 30 seconds); both hands (patients use both hands simultaneously to place as many pins as possible down both rows); right + left + both hands (mathematical sum calculation of the above scores); assembly (patients use both hands simultaneously while assembling pins, washers and collars within 60 seconds) [30].

Descriptive statistics were used for all the items investigated. Statistical analysis was performed using the Statistical Package for Social Science version 20.0 for Macintosh (SPSS Inc., Chicago, IL, USA).

## Results

All subjects completed the treatment protocol (10 training sessions). No adverse events occurred during the study. Demographics are detailed in Table 1.

**Table 1:** **Demographic and clinical characteristics of patients**

	Subject 1	Subject 2	Subject 3
**Age** (years)	69	64	80
**Sex**	Female	Female	Female
**Time since stroke onset** (months)	13	4	9
**European Stroke Scale** (0–100)	80	69	41

Changes in test performance following treatment were observed at the endpoint evaluation as follows. Line Crossing test: all patients showed improved performance (6.7%, 89.5% and 80% increase in lines crossed) with two patients reaching normal performance levels. Bells test: one patient improved performance (50% increase), while one patient showed no change and one patient declined (−10.3% change); no patient reached normal performance levels. Sentence Reading test: all patients showed improved performance (800%, 57.1% and 42.9% increase in number of sentences read), with no patient reaching normal performance level. Saccadic Training: all patients showed improved performance (−62.8%, −15.5% and −9.7% change of the left hemifield reaction time). Sustained Attention to Response Task: all patients showed improved performance (−20.5%, −5.8% and −10% change of the reaction time) with two patients reducing also incorrect responses (−42.9% and −73.3%) and one patient increasing them (9.1%). Purdue Pegboard test: all patients showed improved performance (100%, 27.3% and 75% change in the left + right + both hands sub-item score). Table 2 presents the raw data and percent changes in performance measured at baseline and endpoint evaluations for each subject.

**Table 2:** **Changes in outcome measures following robot-assisted hand training**

Outcome measures	Subject 1			Subject 2			Subject 3		
	Baseline	Endpoint	% Change	Baseline	Endpoint	% Change	Baseline	Endpoint	% Change
**Line Crossing Test** (0–36)	30	32	6.7	19	36	89.5	20	36	80.0
(An omission of 2 lines is pathologic)									
Left	12	14	16.7	6	18	200.0	10	18	80.0
Right	18	18	0.0	13	18	38.5	10	18	80.0
**Bells Test** (0–35)	29	26	−10.3	20	30	50.0	21	21	0.0
(A difference of 5 between right and left is pathologic)									
Left	11	8	−27.3	4	12	200.0	9	8	−11.1
Right	17	17	0.0	15	17	13.3	11	12	9.1
**Sentence Reading Test** (0–12)	0	8	800.0	7	11	57.1	7	10	42.9
(A score <12 is pathologic)									
**Saccadic Training**									
Left (msec)	7252	2698	−62.8	2411	2038	−15.5	5726	5169	−9.7
Right (msec)	2575	2054	−20.2	1062	1446	36.2	3330	5541	66.4
Asymmetry (%)	−47.59	−13.55	−71.5	−38.84	−16.99	−56.3	−26.46	3.47	−113.1
**Sustained Attention to Response Task**									
Reaction time (msec)	511	406	−20.5	452	426	−5.8	612	551	−10.0
Incorrect responses (0–24)	11	12	9.1	14	8	−42.9	15	4	−73.3
**Purdue Pegboard Test** (number of correctly placed pins)									
Left hand	2	1	−50.0	4	5	25.0	0	0	0.0
Right hand	1	2	100.0	12	13	8.3	4	7	75.0
Both hands	0	3	300.0	6	10	66.7	4	7	75.0
Left hand + right hand + both hands	3	6	100.0	22	28	27.3	8	14	75.0
Assembly	1	1	0.0	4	5	25.0	3	4	75.0

## Discussion

The main aim of this case series study was to investigate the effects on hemispatial neglect of a training program based on contralesional robotic limb activation in three patients with stroke. Our findings indicate that robot-assisted left (contralesional) hand training may improve not only visuospatial exploration (as measured by the Line Crossing test, the Bells test, the Saccadic Training and the Sentence Reading test) and attention (as measured by the Saccadic Training and the Sustained Attention to Response Task) but also speed to execute gross movement of the arm, hand and fingers, as well as fingertip dexterity (as measured by the Purdue Pegboard test) in stroke patients with hemispatial neglect.

Our results are in keeping with previous findings on the effectiveness of contralesional limb activation for reducing the level of impairment and disability in patients with hemispatial neglect due to stroke [10–15]. Robertson and North reported that left (contralesional) arm activation produced beneficial effects in a patient with left visual neglect and that active finger movements of the left (contralesional) hand in left (contralesional) hemispace significantly reduce neglect as compared to right (ipsilesional) hand movements or passive visual cueing [10–12]. Gainotti and colleagues found a significant reduction in the severity of neglect in a sample of 7 patients with right brain damage after left (contralesional) hand movements involving the left (contralesional) side of space [13]. In a more recent study, Pitteri and colleagues evaluated, for the first time, the effects of contralesional limb activation treatment alone and in combination with contralesional arm vibration on a patient with left neglect consequent to hemorrhagic stroke in the right cerebral hemisphere [14]. They noted an improvement on the Bells test following the combined application of limb activation and arm vibration as compared to the application of limb activation alone [14]. Our findings on the effects of passive, robot-assisted hand training contrast with those of Robertson and North, who failed to observe a reduction of neglect when the left (contralesional) hand was moved passively [12], but are shared by those of Frassinetti and colleagues, who, applying contralesional hand passive movement according to the entity of proprioceptive signals specifying left hand position, reported an improvement in hemispatial neglect in 8 patients with right brain damage [15].

Our results can be interpreted according to the “premotor model” that suggests the presence in the brain of multiple and dissociable frames of spatial reference (personal, peri-personal and extra-personal) that interact to form a system which is essential for purposeful visuospatial exploration [16, 30]. Accordingly, activation of a limb in the homologous hemispace (i.e., activation of the left hand in the left hemispace) leads to a reciprocal enhancement of the personal and peripersonal perceptuomotor neural maps and produces a coherent spatial reference system [17, 31]. From a rehabilitative point of view, contralesional limb movement is thought to activate the damaged hemisphere and counteract the inhibitory competition from the undamaged hemisphere, leading to a reduction of neglect symptoms in patients with hemispatial neglect after stroke.To the best of our knowledge, this is the first study to investigate the effects of robotic limb activation on hemispatial neglect in people with stroke. The three subjects received robot-assisted hand training on the Gloreha® machine that provides computer-controlled, repetitive, passive mobilization of the fingers (Figure 1). This is relevant for rehabilitation, given that, unlike other types of conventional physical therapy, the Gloreha® machine allows to initiate already at the bedside early treatment with a high number of repetitions of passive movement sequences in combination with real-time 3D animation of hand movements shown on a monitor positioned in the patient’s contralesional visual field (extrapersonal space). Such a treatment setting may further enhance the spatial reference system disrupted in patients with hemispatial neglect by activating the extrapersonal perceptuomotor neural maps when patients watch a video animation of their hand movements (see the “premotor model” described above). However, we cannot exclude that asking the subjects to name their moving fingers (either by vision or proprioception) may have further increased awareness of the left hand and thus influenced the effects of robot-assisted passive limb activation observed in this study.

Some caution is warranted when interpreting our results, as the responses to the intervention were variable, especially for the Bells test. Though such heterogeneity is in line with the inherent variability seen in attentional disorders after stroke [32, 33], the observed changes might have been due to a placebo effect or fluctuating clinical conditions in the absence of multiple baseline measurements. Additional limitations of this study are the small sample size and the lack of a control group. No conclusions can be drawn about the role of robotic training in patients with hemispatial neglect.

## Conclusions

Robot-assisted hemispatial neglect therapy might be useful in patients with stroke. Larger-scale investigations and randomized controlled trials are needed to confirm our preliminary findings and to establish what benefit people with hemispatial neglect can get from robotic hand training, what treatment regimen should be adopted (i.e., exercise protocol, treatment duration) and which is the best rehabilitative practice (i.e., conventional versus robotic training).

## Disclosures

The authors received no financial support for the research or authorship of this article.

No commercial party having a direct financial interest in the results of the research supporting this manuscript has or will confer a benefit on the authors or on any organization with which the authors are associated.

## Authors’ original submitted files for images

Below are the links to the authors’ original submitted files for images. Authors’ original file for figure 1

## Abbreviations

FES: Functional electrical stimulation.
