# Supplementary figures and images for: Effects of contralesional robot-assisted hand training in patients with unilateral spatial neglect following stroke: a case series study
Source: J Neuroeng Rehabil. 2014 Dec 5;11:160. doi: 10.1186/1743-0003-11-160 (PMC4271413; doi:10.1186/1743-0003-11-160)

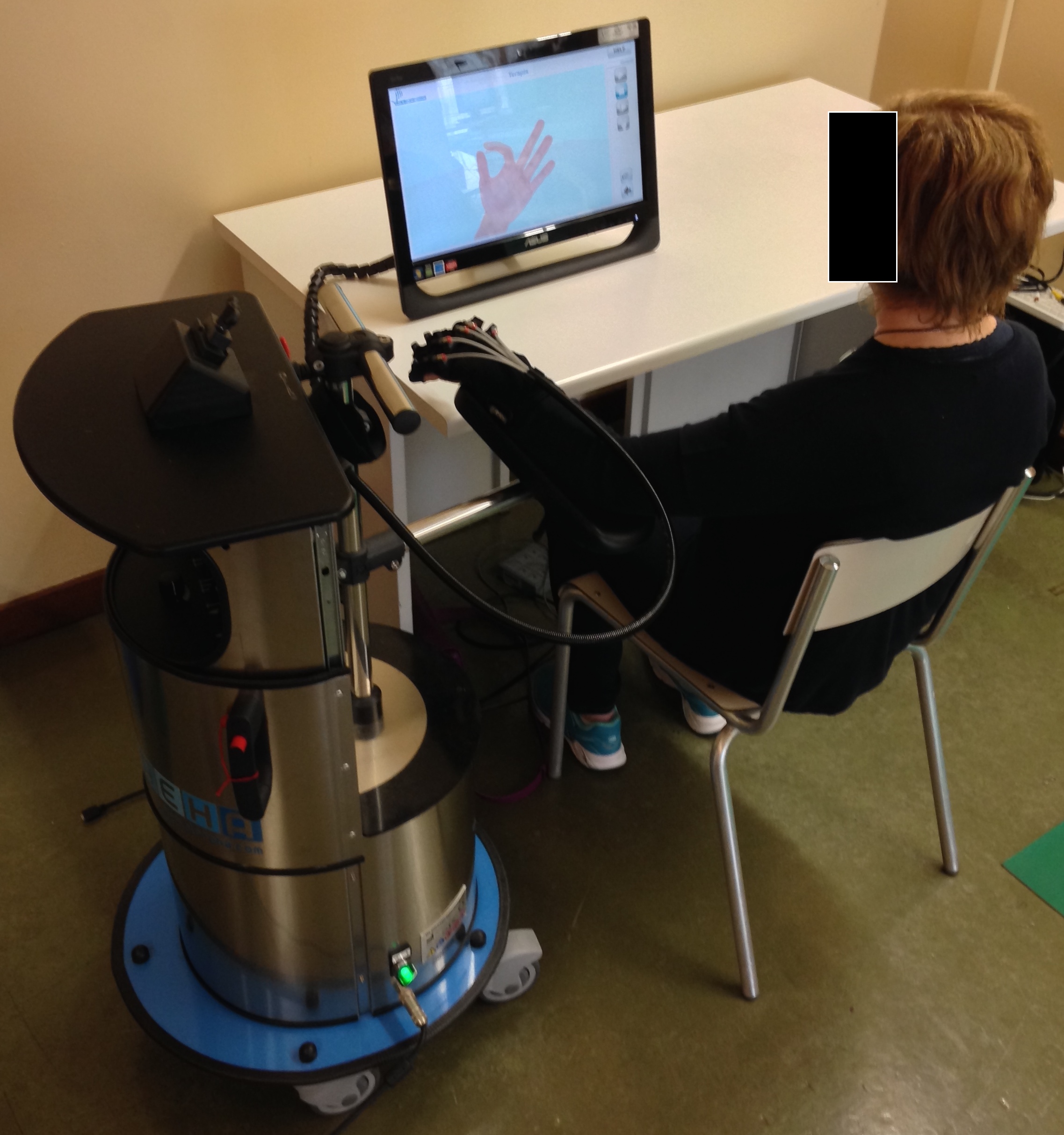

Supplement: Supplementary file 1 — Authors’ original file for figure 1 [file 12984_2014_680_MOESM1_ESM.jpeg]
